# On the importance of including both sexes in animal studies – insights from home‐cage monitoring

**DOI:** 10.1002/brv.70184

**Published:** 2026-05-13

**Authors:** Maša Čater, Adam Dušek, Aleksandra Bartelik, Silvia Mandillo, Elisabetta Golini, Mara Rigamonti, Rasneer Sonia Bains, Hamish Forrest, Marion Rivalan, Melissa Long, Anna Kiryk, Lars Lewejohann, Nuno Henrique Franco

**Affiliations:** ^1^ Department of Animal Science, Biotechnical Faculty University of Ljubljana Groblje 3 Domžale 1230 Slovenia; ^2^ Department of Ethology, Institute of Animal Science Přátelství 815 Prague 10400 Czech Republic; ^3^ International Clinical Research Center St. Anne's University Hospital Brno Pekařská 53 Brno 602 00 Czech Republic; ^4^ Institute of Biochemistry and Cell Biology IBBC National Research Council CNR Via E. Ramarini 32 Monterotondo (Rome) 00015 Italy; ^5^ Tecniplast S.p.A Via I Maggio 6, 21020 Buguggiate Italy; ^6^ Mary Lyon Centre at Medical Research Council Harwell Oxfordshire OX11 0RD UK; ^7^ Institute of Neuroscience Paris‐Saclay CNRS, Université Paris‐Saclay Saclay France; ^8^ Animal Behaviour Phenotyping Facility Charité – Universitätsmedizin Chariteplatz 1 Berlin 10117 Germany; ^9^ Laboratory of Preclinical Testing of Higher Standard, Neurobiology Center, Nencki Institute of Experimental Biology, Polish Academy of Science 3 Pasteur Street Warsaw 02‐093 Poland; ^10^ Institute of Animal Welfare, Animal Behaviour and Laboratory Animal Science, Department of Veterinary Medicine Freie Universität Berlin Königsweg 67 Berlin 14163 Germany; ^11^ German Centre for the Protection of Laboratory Animals (Bf3R) German Federal Institute for Risk Assessment (BfR) Max‐Dohrn‐Str. 8‐10 Berlin 10589 Germany; ^12^ i3S ‐ Instituto de Investigação e Inovação em Saúde Universidade do Porto Rua Alfredo Allen Porto 208 4200‐135 Portugal

**Keywords:** sex differences, animal studies, animal research, preclinical research, biomedical research, behaviour, home‐cage monitoring

## Abstract

A review of behavioural studies using home‐cage monitoring (HCM) systems revealed that over 61% of studies used only male subjects, with only 24% including both sexes, despite evidence of substantial behavioural differences between male and female animals. This bias could influence the outcomes of biomedical research. This preference for male subjects has introduced a significant bias in preclinical animal studies, compromising the generalisability of research findings and potentially overlooking important sex differences in disease manifestation and response to therapies. This review highlights such sex differences in laboratory rodent behaviour, as observed through HCM systems. HCM provides an automated method for observing the behaviour of undisturbed animals in a familiar environment, offering valuable insights into sex‐specific differences in behaviour and disease progression. In particular, we emphasise how this technology revealed sex differences in spontaneous activity and behaviour in models of neurodegenerative diseases, while demonstrating that, contrary to common belief, female rodents are not more variable than males in their behaviour. We further discuss the reasons underlying the prevalent sex bias in research and advocate for the inclusion of both sexes in neuroscience and behavioural studies, to enhance the generalisability and informative value of preclinical research, avoid unethical waste of animals and resources, and thereby support the Three Rs principles (Replacement, Reduction, and Refinement of animal use).

## I. INTRODUCTION

In recent years, it has become apparent that most preclinical studies are carried out on animals of only one sex, in most cases in males. This is a significant problem, as sex differences affect significantly the generalisability of conclusions (see Nunamaker & Turner, 2023), raising the urgent need to address this issue in all fields of research using animal models. This includes research using home‐cage monitoring (HCM), a promising approach to obtain a comprehensive overview of laboratory animal behaviour through automated systems (Bartelik *et al*., 2024). A recent systematic review (Kahnau *et al*., 2023) revealed that the common single‐sex bias observed in biomedical research is also found in HCM studies. Of the 521 publications from 1974 to 2020 included, more than 61% reported using only males, 12% only females, and 24% used both sexes, with 3% not reporting the sex used. As a general positive trend, the use of both sexes has gradually increased, but even in 2020, amounted to only 30% of studies. Applying the same search query for 2021 and 2022 on *PubMed*, we find that for those 2 years the use of both sexes has increased to 33%, with 46% of studies still only examining males.

Most biomedical experiments lack data from female subjects, including both animal research and cell/tissue culture studies (Plevkova *et al*., 2020; Zucker & Beery, 2010). Several reasons for using laboratory animals of a given sex have been described in the literature, which are sometimes contradictory. In most cases, male animals are preferred, with the most common explanation being that, presumably, the oestrus cycle in females leads to large variations, making it difficult to draw consistent conclusions.

According to Zucker & Beery (2010), considerations on this topic began at the start of the 20th century. Wang (1925) showed that the locomotor activity of female rats (*Rattus norvegicus*) changes with their oestrous cycle. Slonaker (1924) found rhythmic changes in voluntary activity in female rats, but not in male rats, during longitudinal observations in female and male rats in revolving cages. He extended his observations by examining the dependencies of the observed changes on diet and on animal age. Adult, sexually mature female rats were significantly more active approximately every 4 days. This regularly increased activity disappeared in old females at the end of the reproductive period. Inadequate nutrition also affected the disappearance of the rhythmic increase in activity in female rats.

Based on this information, Brobeck, Wheatland & Strominger (1947) examined the regulation of energy exchange in connection with oestrus, dioestrus and pseudopregnancy in female rats by also observing food intake, body mass and body temperature of the animals. They were interested in investigating the relationship between the oestrus cycle and activity of the hypothalamus, which influences body temperature regulation, spontaneous motor activity, food intake and body mass. In their experiment, hyperactivity during oestrus was associated with reduced food intake, body mass loss and lower body temperature. Brobeck *et al*. (1947) argued that these periodic changes in energy exchange processes are due to either the hypophysial or the ovarian cycle.

Interestingly, knowledge of the effects of oestrus cycle variation on female test results did not reinforce the belief that it was necessary to conduct research with both sexes. On the contrary, Lewis (1960) suggested minimising experimental variation by using only one sex in experiments. This paradoxically reinforced the belief that a sex that was less ‘variable’, namely the male, was the most appropriate to use. Clinical research has also promoted a thesis of a need to ‘protect’ young women by conducting studies mainly on men (Docherty *et al*., 2019). Mogil & Chanda (2005, p. 1) proposed that ‘the major reason for the overwhelming use of male subjects is simply inertia, but inertia reinforced by the following assumption: that the oestrous cycle of female rodents imposes additional variability onto experiments’. Despite numerous studies documenting differences between the sexes due to the influence of hormones and the relationships between the hormonal and nervous systems, and despite experiments showing differences in behaviour (Broverman, Klaiber & Kobayashi, 1968; Terranova, Laviola & Alleva, 1993; Võikar *et al*., 2001), the approach of including only males has not been abandoned. However, these early and now rather dated studies have been revisited in light of more recent evidence, which suggests that the oestrous cycle may not contribute as much variability as once assumed. Indeed, Levy *et al*. (2023) demonstrated that, in contrast to female rats, the oestrous cycle in female mice (*Mus musculus*) had only a minimal effect on exploratory activity. Instead, individual behavioural differences among subjects played a more significant role (Levy *et al*., 2023). These findings indicate that observed behaviours were more strongly related to the unique characteristics of each animal than to generalised sex‐related traits. Furthermore, meta‐analyses aggregating hundreds of behavioural indicators confirmed that overall behavioural variability in females is not greater than in males at any stage of the oestrous cycle. This conclusion was consistent across studies conducted on rats (311 articles analysed) (Becker, Prendergast & Liang, 2016), mice (293 articles) (Prendergast, Onishi & Zucker, 2014), and combined analyses of both species (263 articles) (Kaluve, Le & Graham, 2022).

In male subjects, where behaviour was often attributed to hierarchical or dominance factors, Levy *et al*. (2023) observed greater variability not only among different males but also within the same individual across different contexts and over time. This finding suggests that male behaviour in research settings is not as uniform and predictable as once assumed. In fact, male behaviour may be less stable over time compared to female behaviour.

There are also studies in which the researchers use only female animals. We exclude here studies on female‐specific diseases (e.g. ovarian cancer). The most common explanation for the use of only female animals as research subjects is male aggression (Franco, Correia‐Neves & Olsson, 2012), which has been shown to have a direct impact on the quality and reproducibility of the research. Male aggression causes greater variability (Gaskill, 2014; Weber *et al*., 2022), contradicting the belief that male mice are less‐variable research subjects.

In response to mandates to include both sexes in preclinical research, some commentators have expressed the opinion that this could introduce conceptual and empirical errors into the research (Richardson *et al*., 2015), while others have argued that studying sex differences should also take into account sex‐specific social and environmental factors (DiMarco *et al*., 2022). However, growing evidence shows that including both sexes does not just add variability or reduce reproducibility. Instead, it reveals biologically relevant differences that would otherwise remain undetected when research is restricted to a single sex. These sex‐specific effects can be systematically addressed through appropriate experimental design and statistical modelling. Thus, rather than introducing error, the inclusion of both sexes enhances both the rigour and the translational value of preclinical research.

We argue that, unless for obvious reasons such as the study of sex‐specific traits (e.g. specific male or female hierarchical dynamics) or diseases (e.g. testicular cancer), carrying out studies on just one sex is rarely justifiable. And indeed, in the aforementioned systematic review on HCM research (Kahnau *et al*., 2023) most studies excluding females did not discuss the reasons why. Among the few reasons given were the traditional concern about the influence of oestrogen on behaviour and the cyclic fluctuations associated with oestrus. However, as a rule, data were not collected and excluded retrospectively, but rather females were not considered from the outset. Any justification, when present, referred exclusively to the literature.

Such perspectives may lead to much abbreviated statements, which on closer inspection, can neither be derived from the original literature nor actually be generalised. For example, one paper claimed that fear conditioning was only conducted with males, ‘since oestrogen levels and the oestrus cycle phase can influence memory and fear processing’ (Hickey *et al*., 2008, p. 282), but while the literature does show that such influencing factors exist, it in no way suggests that fear conditioning should not be conducted with female subjects.

## II. BIOLOGICAL BASIS OF SEX DIFFERENCES

In most mammals, including the mouse and the rat, sex is genetically determined by sex chromosomes (XX for female or XY for male). However, sexual differentiation of brain and behaviour depends on the interplay between genetic, hormonal, and epigenetic mechanisms acting during a critical perinatal period (Forger, 2016), as well as the physical and social environment (Cox & Rissman, 2011; Lin *et al*., 2011). Sex differences in the brain are established and maintained epigenetically by DNA methylation, methyl‐binding proteins and histone and chromatin modifications (Nugent & McCarthy, 2011). These processes are under the control of sex hormones, can differentiate by and within specific brain regions, and are responsible for sex differences in various specific behaviours (McCarthy & Nugent, 2013; Zuloaga *et al*., 2020) and susceptibility to a spectrum of neurological and psychiatric disorders (Qureshi & Mehler, 2010). The differentiation of sex‐specific behaviours is caused by the enzymatic conversion of testicular testosterone (T) to dihydrotestosterone (DHT) by 5α‐reductase and to oestradiol (E2) by aromatase in the brain (Filova *et al*., 2013). The activity of these two metabolic pathways influences gene expression in brain cells *via* androgen and oestrogen receptors (Menger *et al*., 2010). However, some perinatally induced epigenetic changes may not be permanent. If an adult animal is exposed to high levels of hormones of the opposite sex over a prolonged period, this can lead to a sex reversal in behaviour (McCarthy, 2019).

Interindividual variation within sexes refers to differences among organisms of the same sex within a species (Graham, 2023). Such variation can be influenced by genetic, epigenetic, and environmental factors and can contribute significantly to phenotypic differences in both animal (Löscher, 2024) and human (Khodursky *et al*., 2022) populations. In polytocous (litter‐bearing) mammals, such as mice and rats, individual differences are largely shaped by intrauterine position, which affects prenatal exposure to opposite‐sex hormones and results in varying degrees of brain and behavioural masculinisation or feminisation (Ryan & Vandenbergh, 2002; vom Saal, 1981). Comparable effects on sexually dimorphic traits and health outcomes have been reported in human twins (Ahrenfeldt *et al*., 2020; Vandenbergh, 2009). Sexually dimorphic brain regions are further influenced by maternal gestational hormones (Miranda & Sousa, 2018), with sex‐specific effects linked to neuropsychiatric disorders such as dementia (Luo *et al*., 2020) and autism spectrum disorder (ASD; Firestein *et al*., 2022). However, the mechanisms by which natural variation in brain organisation shapes individual behavioural phenotypes within each sex remain to be fully delineated (see e.g. Kundakovic & Tickerhoof, 2023). The use of HCM systems appears to be a promising approach for precisely evaluating this intrasexual variation in preclinical research, in which capturing the full spectrum of biological responses to treatment is essential (Kahnau *et al*., 2023; Voikar & Gaburro, 2020).

In male subjects, where behaviour was often attributed to hierarchical or dominance factors, Levy *et al*. (2023) observed greater variability not only among different males but also within the same individual across different contexts and over time. This finding suggests that male behaviour in research settings is not as uniform and predictable as once assumed. In fact, male behaviour may be less stable over time compared to female behaviour.

## III. SEX DIFFERENCES IN RODENT ACTIVITY, BEHAVIOUR, AND OTHER PARAMETERS

### (1) Activity

Due to rodents being prey for multiple predators, a rodent's activity within the home cage or test arena is driven by two opposing factors: motivation for exploration and fear of the unknown (Sturman, Germain & Bohacek, 2018). Laboratory mice show horizontal and vertical locomotor activities as well as many activities related to cage bars, such as gnawing on bars and climbing on the cage lid. The latter has been described as a form of motor routine, an attempt to escape the cage environment, or a repetitive stereotypic behaviour (Nevison, Hurst & Barnard, 1999). This type of activity has also been associated with a coping response in mice that helps them reduce the stress caused by captivity (Mason, 1991). Activity is therefore an important parameter to observe when studying animal behaviour. A reduction or absence of cage‐climbing activity reflects the emotional state of the animal and is therefore associated with some pathologies such as increased anxiety‐like behaviour in mice (Pietropaolo *et al*., 2007).

**Fig. 1:**
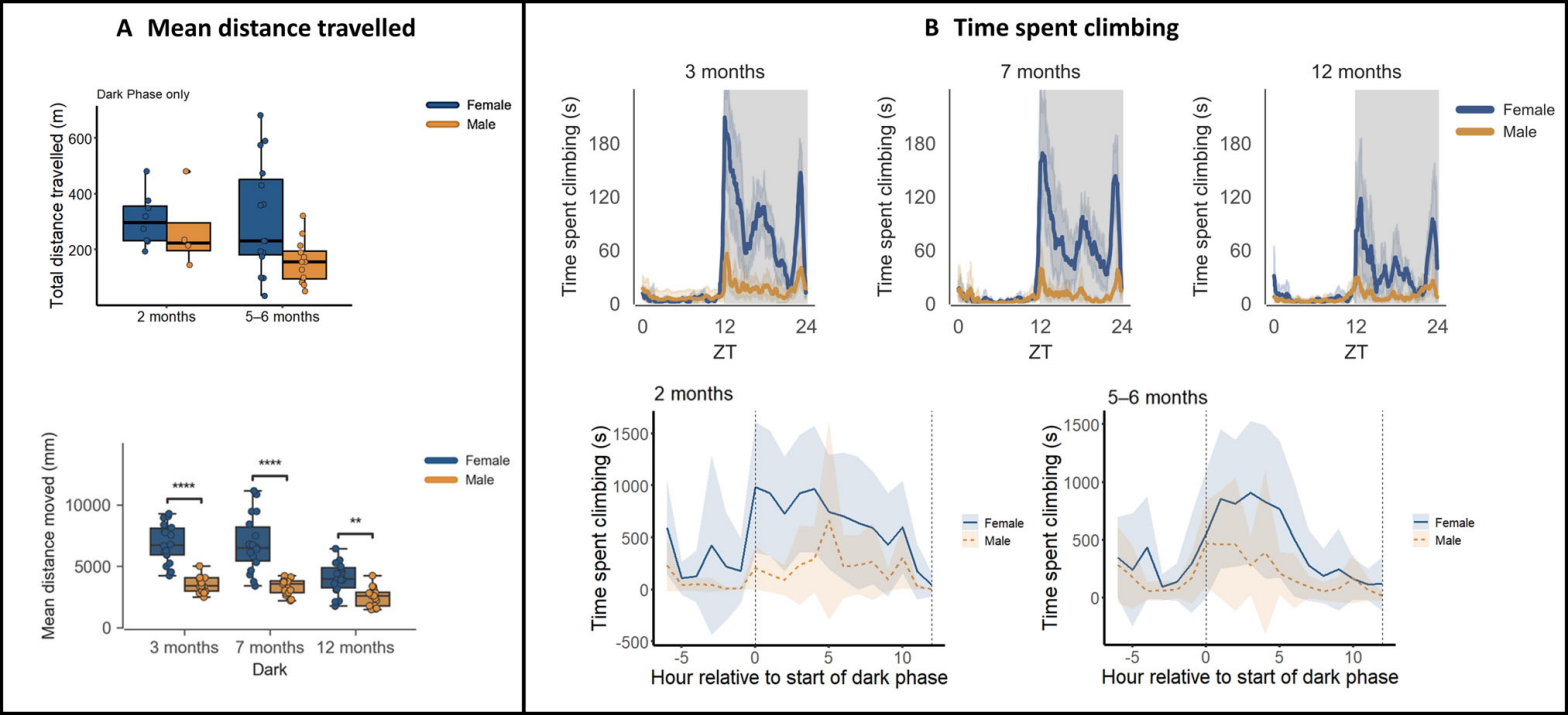
Distance travelled (A) and climbing activity (B) during the dark phase vary according to sex during passive home cage monitoring (HCM). Cage‐lid climbing in B perfectly recapitulates the differences between male and female activity seen for cage floor activity in A using a different home‐cage system. (A) Top: mean distance travelled during the dark phase in the Home Cage Analyser (HCA) (three data points per cage for 3 days of recording; *N* = 36 mice: 18 males and 18 females, housed in cages of 3). A linear mixed‐effects model was used to account for repeated‐measures and least‐squares means estimated to calculate adjusted *P* values for levels of factor combinations. **P < 0.05, ****P < 0.0001. Data from Bains *et al*. (2023); provided as supporting information in Data S1. Bottom: total distance travelled by singly housed mice during one dark phase in the Home Cage Scan (HCS) system (2 months old *N* = 8 females, *N* = 5 males; 5–6 months old *N* = 16 females, *N* = 12 males). Wilcoxon test between sexes at age 2 months, n.s. (*W* = 20, *P* = 0.5697) and at age 5–6 months (*W* = 135, *P* = 0.02814). Box plots show the interquartile range (boxes), median (line), and whiskers at the 99% limits. Data are from Rodriguez de los Santos *et al*. (2021) for age 2 months and from Stolting *et al*. (2023) and the ABPF (unpublished data) for age 5–6 months; provided as supporting information in Data S1. (B) Top: differences in time spent climbing (in seconds) over zeitgeber time (ZT; time in hours after light onset) between female and male cages for mice aged 3 (*N* = 6, *P* < 0.0001), 7 (*N* = 6, *P* < 0.0001) and 12 (*N* = 6, *P* < 0.0001) months during recording sessions in the HCA system, binned into 6‐min time bins and averaged over a 24‐h period. The line represents time spent climbing across cages of a sex group; shaded error bands represent 95% confidence intervals. Data from individual mice within a cage were summed to produce one time series per cage. Grey background shading represents the dark phase. Data from Bains *et al*. (2023). Bottom: time spent climbing in singly housed female and male 2‐ and 5–6‐month‐old mice (2 months old *N* = 8 females, *N* = 5 males; 5–6 months old *N* = 16 females, *N* = 12 males) during recording session in the HCS system, binned per hour. Lines represent averaged time spent climbing per sex and time; shaded ribbon represents ±1SD. Vertical lines at hours 0 and 12 indicate start/end of darkness. Markov chain Monte Carlo generalised linear mixed models (MCMCglmms) with animal and cage as random factors were used for between‐sex comparisons at age 2 months (*P*_MCMC_ = 0.0472, posterior mean = −362.094, 95% CI = −702.558 to −6.211); and at age 5–6 months, *P*_MCMC_ = 0.00315, posterior mean = −221.75, 95% CI = −377.28 to −56.54). Data are from Rodriguez de los Santos *et al*. (2021) for age 2 months and are from Stolting *et al*. (2023) and the ABPF (unpublished data) for age 5–6 months; provided as supporting information in Data S1.

While meta‐research has confirmed that the inclusion of female animals in behavioural studies does not lead to increased variability in activity (Prendergast *et al*., 2014; Fritz, Amrein & Wolfer, 2017), HCM research has shown significant differences between female and male mice. Borbélyová *et al*. (2019) observed sex differences using C57BL/6NTac mice in horizontal locomotor activity using the LABORAS system, which revealed that male mice travelled shorter distances and moved more slowly than females; females were also more active in grid climbing than males. This finding is in agreement with Dere *et al*. (2018), who reported that females in general exhibit higher spontaneous locomotor activity than males in the home‐cage system IntelliCage, while Fuochi *et al*. (2021) found that sex differences in activity are strain specific, using a Digital Ventilated Cages (DVC) monitoring system. The latter studied the spontaneous locomotor activity of two inbred mouse strains (C57BL/6JNCrl and BALB/cAnNCrl) and one outbred mouse strain [CRL:CD1(ICR)], showing that C57BL/6JNCrl and CRL:CD1(ICR) strains exhibited sex‐related differences in activity, with females displaying higher average locomotor activity from prepuberty to adulthood than males. CRL:CD1(ICR) mice also had the highest average activity recorded over extended periods. However, BALB/cAnNCrl males and females displayed similar motor phenotypes. Using the Home Cage Analyser (HCA) system to record spontaneous cage base activity and cage‐lid‐climbing activity of group‐housed animals, Bains *et al*. (2023) showed that sex differences persist as C57BL/6J males and females age. Figure 1 shows part of these data focusing on the horizontal and vertical (climbing) activity of the control C57BL/6J groups of mice (see online Supporting Information, Data S1), alongside data collected at the Animal Behaviour Phenotyping Facility of Charité University [previously unreported data from Stölting *et al*. (2023) and Rodríguez de Los Santos *et al*. (2021), see Data S1], in mice of the same strain but using a different home‐cage monitoring (HCM) system, namely HomeCageScan (HCS). In the HCS system, singly housed C57BL/6J males and females were recorded overnight. Figure 1A describes the distance travelled in the dark phase captured using these two different home‐cage systems, the boxplot in the top panel describes mean distance travelled during the dark phase in group‐housed mice, recorded longitudinally in the HCA system, while the bottom panel describes the total distance travelled in the dark phase for singly housed animals, recorded longitudinally in the HCS system. A statistically significant difference in cage floor, as well as cage lid activity is seen between male and female C57BL/6J mice that persists with age and is observed in both systems. Figure 1B describes the cage‐lid‐climbing activity in both systems; the top panel shows time spent climbing (s) in group‐housed mice recorded in the HCA system and the bottom panel shows time spent climbing (s) in singly housed animals recorded in the HCS system. There was persistently lower cage activity, whether measured as mean distance moved or time spent climbing, in males across every time point and in both systems.

Independently of the HCM system used, an inherent difference between male and female vertical and horizontal activity was found, which persists with age, for mean distance moved from 3 months old (not statistically significantly different for total distance travelled at 2 months), and from 2 months old for climbing activity, until 12‐months‐old in both singly and group‐housed mice. These results, consistent across independent laboratories using different HCM systems, confirm that the observed sex differences in spontaneous activity are not an artefact of the recording system used. Moreover, results from HCM systems are not confounded by emotional responses, which can be triggered in standard ‘out‐of‐cage’ testing paradigms. These results highlight the importance of recording data from both sexes.

Advanced HCM technology allows large‐scale automated monitoring of the animal in its home cage, enabling researchers to gain a deeper insight into normal behaviour patterns using long‐term monitoring. An illustrative example shows how regular handling and cage cleaning can affect female and male mice differently. A study using C57BL/6J mice and a DVC system reported that cage changing, as part of a regular cleaning protocol, affected both female and male activity (Pernold *et al*., 2019). Changes in baseline activity patterns were observed over an extended period. Male mice showed a longer lasting impact on average daytime activity after a cage change than female mice, possibly due to more complex re‐establishment of hierarchy and territory control among males than females. Sex‐related and strain‐specific exploratory and marking behaviour was also reported by Fuochi *et al*. (2021).

**Fig. 2:**
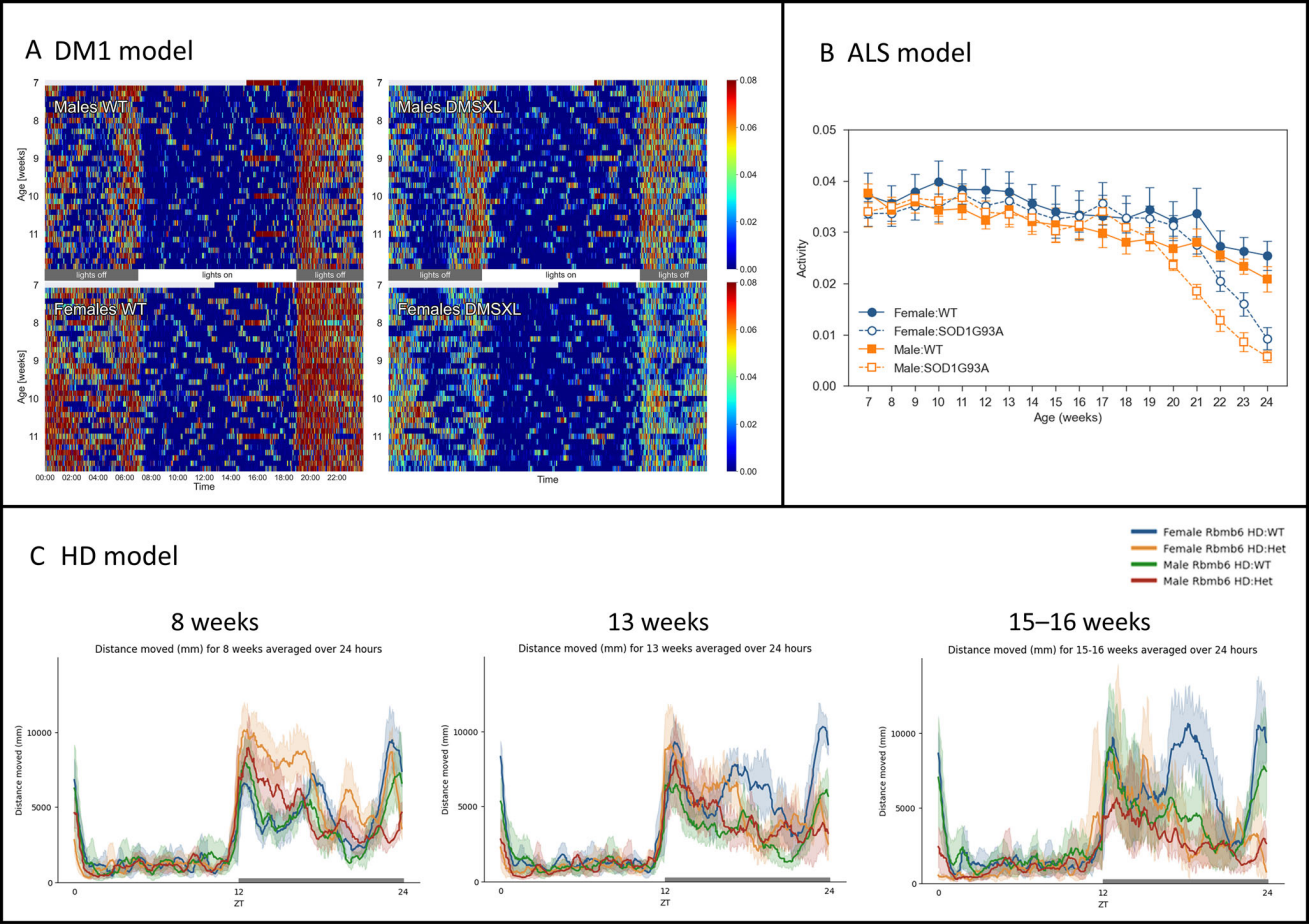
Motor activity and sex differences in three models of neurodegenerative diseases. (A) Heatmap of activity in male and female myotonic dystrophy type 1 model (DMSXL) mice compared with wild‐type (WT) mice. Light and dark periods are shown by the horizontal bar across the centre (lights on: 07:00; lights off: 19:00). A compilation of results from the continuous monitoring of cage activity of 2 mice per cage for about 5 weeks is shown. Data are from Golini *et al*. (2023). (B) Continuous activity phenotype progression in an amyotrophic lateral sclerosis (ALS) model SOD1G93A in male and female mice compared with WT mice using the digital ventilated cage (DVC) system. Home‐cage activity was recorded continuously using the DVC system for almost 5 months. Average weekly activity (±SEM) during the night time. Number of cages (2 mice per cage): Males WT = 8; SOD1G93A = 9; Females WT = 11; SOD1G93A = 10. Data are from Golini *et al*. (2020). (C) Progression of Huntington's disease (HD) model (Rbmb6 HD:Het) recorded at three time points in the same mice (*N* = 108; *N* HD Females = 33, *N* HD males = 27, *N* WT for each sex = 24) using the Home Cage Analyser (HCA) system. ZT, zeitgeber time. Line represents mean distance over time across cages for a sex and age group; shaded area represents 95% confidence interval. Data are from Bains *et al*. (2023).

HCM systems allow differences between genotypes as well between sexes to be monitored in home cages for long periods without disturbing the animals; such features are of particular relevance when studying models of severe and progressive neurodegenerative diseases. Figure 2 shows examples from previously published data on three different models of neurodegenerative diseases that highlight potentially important sex differences and pinpoint some aspects of activity that are only revealed using HCM tools. In Fig. 2A, a heatmap of cage activity recorded using the DVC system shows the highly reduced activity (especially between 19:00 and 7:00) of DMSXL mice compared with wild‐type (WT) mice. DMSXL mice are a model of myotonic dystrophy (DM1; Golini *et al*., 2023). Note also that the activity of WT mice is greater in females compared to males (more red colour during the dark phase). Heatmaps are thus useful when long‐term data are acquired. For this particular neuromuscular disease model, we did not observe any age‐related changes over the period of observation (7–12 weeks of age), but this is not a model of a progressive disease. Figure 2B shows a decline in night‐time activity in SOD1G93A mouse mutants, a model for the severe progressive neuromuscular disease amyotrophic lateral sclerosis (ALS; Golini *et al*., 2020). Disease progression is more pronounced in males and also note that WT female mice (blue lines) are more active than males (orange lines). Similar genotype and sex differences were observed in another mouse model, the B6‐TgN(HD82Gln)81Dbo/H (referred to as Rbmb6 HD in the figure legends), also known as the N171‐82Q model of Huntington's disease (Fig. 2C). Disease progression can be appreciated by comparing activity levels at the three specific age points shown (Bains *et al*., 2023).

**Fig. 3:**
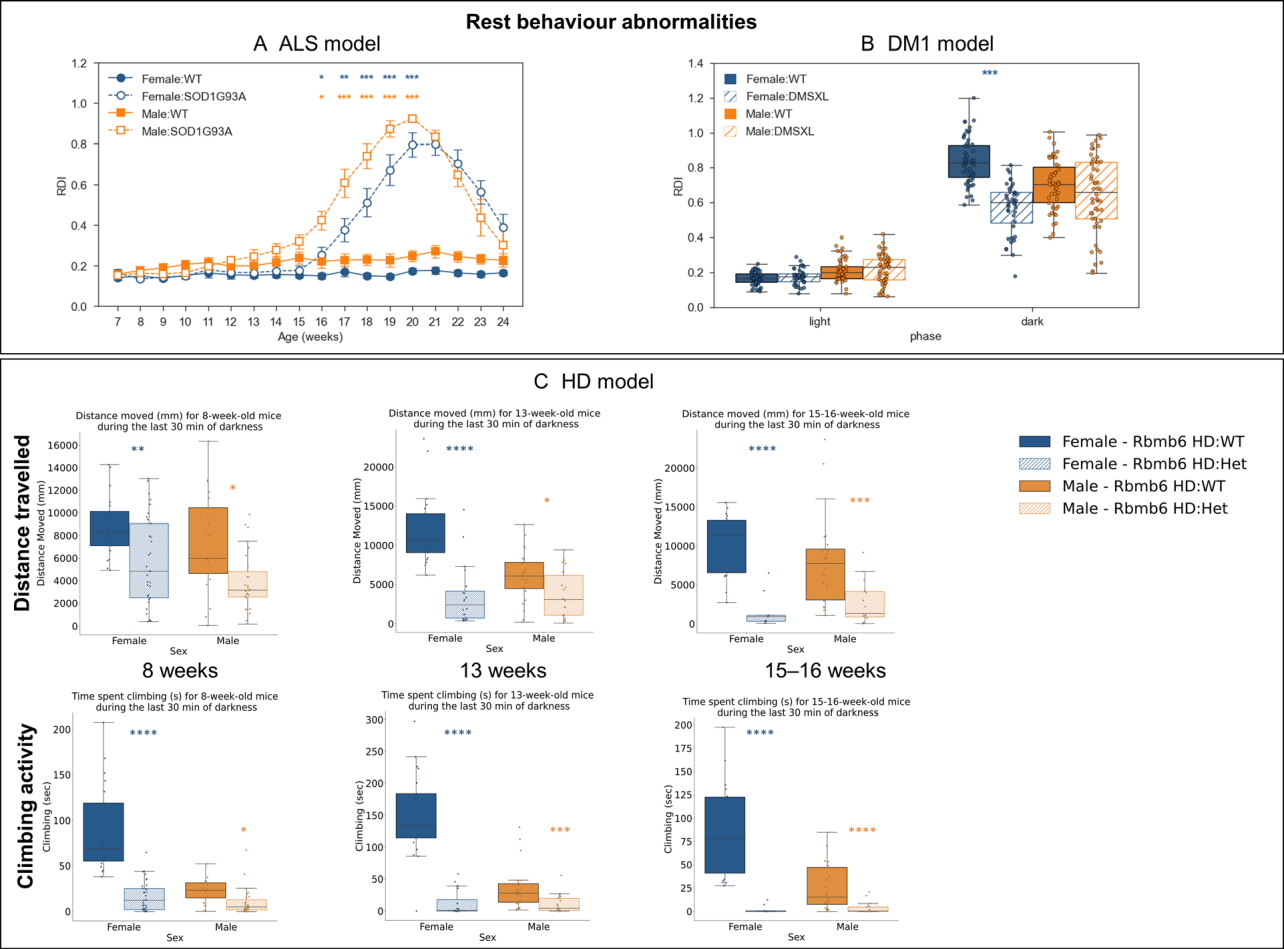
Motor activity and sex differences in three models of neurodegenerative diseases. (A, B) Changes in Regularity Disruption Index (RDI), a measure unique to continuous home cage monitoring (HCM) and recorded in the digital ventilated cage (DVC) system, which can be used as an index of sleep and rest disturbances. (A) Wild‐type (WT) mice compared with an amyotrophic lateral sclerosis (ALS) mouse model (SOD1G93A mice). Data (mean ± SEM) were recorded during the day (resting phase). Number of cages per group (2 mice per cage): Males WT = 8; Males SOD1G93A = 9; Females WT = 11; Females SOD1G93A = 10. *P < 0.05, ** *P* < 0.01, *** *P* < 0.001 WT *versus* SOD1G93A, D/AP (Dubey/Armitage‐Parmar) *post‐hoc* procedure. After age week 20 we could not perform statistical tests since some subjects were missing, resulting in smaller group sizes at subsequent time points (number of cages per group at age week 21: Males WT = 8; Males SOD1G93A = 8; Females WT = 11; Females SOD1G93A = 10; age week 22: Males WT = 6; Males SOD1G93A = 7; Females WT = 8; Females SOD1G93A = 8; age week 23: Males WT = 6; Males SOD1G93A = 7; Females WT = 8; Females SOD1G93A = 7; age week 24: Males WT = 6; Males SOD1G93A = 4; Females WT = 7; Females SOD1G93A = 6. Data are from Golini *et al*. (2020). (B) WT mice compared with a myotonic dystrophy type 1 (DM1) mouse model. Average weekly RDI (±SEM). Number of cages per group (2 mice per cage): Males WT = 10; Males DMSXL = 10; Females WT = 11; Females DMSXL = 8. Bonferroni‐corrected *post‐hoc* tests to compare DMSXL and WT males and DMSXL and WT females [***P < 0.001, nparLD (nonparametric analysis of longitudinal data) test with genotype and week as factors]. The boxes represent the interquartile range (Q1–Q3), the central line indicates the median, whiskers extend to 1.5 × IQR, and individual points denote outliers. Data are from Golini *et al*. (2023). (C) Data recorded by the Home Cage Analyser (HCA) system, for a Huntington's disease (HD) model. Males show low baseline activity in both WT animals and the HD model. Through continuous monitoring of voluntary behaviour over multiple light/dark cycles, HCM makes it possible to resolve detail in the temporal patterns of phenotypic traits by analysis of specific times of interest around the dark to light transition. Top row: boxplot of mean distance moved during the 30 min of darkness within single‐sex cages split by age and genotype (*N* = 108; *N* HD females = 33, *N* HD males = 27, *N* WT for each sex = 24). Distance moved was averaged across the last 30 min of darkness per cage and per day. Three data points per cage (3 days of recording) were modelled using a linear mixed‐effects model to account for repeated‐measures and least‐squares means estimated to return adjusted *P* values of levels of factor combinations. **** *P* < 0.0001. Bottom row: boxplot of mean time spent climbing during the last 30 min of darkness within single‐sex cages split by age and genotype (*N* = 108; *N* HD females = 33, *N* HD males = 27, *N* WT for each sex = 24). Time spent climbing was averaged across the last 30 min of darkness per cage and per day. Three data points per cage (3 days of recording) were modelled using a linear mixed‐effects model to account for repeated‐measures and least‐squares means estimated to return adjusted *P* values of levels of factor combinations. * *P* < 0.05, **** *P* < 0.0001. Data are from Bains *et al*. (2023).

Long‐term automated HCM also allows the detection of subtle changes in motor activity, as well as sex differences. In Fig. 3A, B, rest behaviour abnormalities measured using the Regularity Disruption Index (RDI) are shown for the ALS and DM1 mouse models. This metric quantifies irregular patterns of activity and can be considered as an index of sleep and rest disturbances (Golini *et al*., 2020, 2023). These types of disturbances are common in many neurodegenerative diseases. Disrupted sleep is often observed in ALS patients and daytime sleepiness affects almost 80% of DM1 patients (Boentert, 2019; Subramony *et al*., 2020). Figure 3A (ALS model) shows an increased RDI in SOD1G93A mice, in both males and females, during the day (resting phase), between the ages of 16 and 23 weeks. RDI curves in SOD1G93A mice show a bell‐shaped pattern, with an initial increase followed by a peak and a subsequent decline. This is likely due to progressive neuromuscular deterioration after ~20 weeks of age, which limits mobility and increases inactivity, resulting in lower RDI values. Male transgenic mice show earlier expression of this phenotype compared to females (Golini *et al*., 2020) and ALS has an earlier onset in men (McCombe & Henderson, 2010; Manjaly *et al*., 2010). By contrast, in the DM1 model (Fig. 3B), the RDI is reduced (more sleep) during the dark (active phase) in DMSXL mice, especially in females, suggesting similarity with the daytime sleepiness symptom widely reported in DM1 patients and apparently more severe in women (Golini *et al*., 2023).

We have shown that female and male mice have different levels of baseline activity, and that these differences persist over time (see Fig. 1A). Therefore, when mice are used to model human diseases, e.g. where a change in activity indicates disease onset and is also used for measuring therapeutic efficacy of a treatment, we argue that it is imperative to test both sexes: testing only one sex will result in preclinical work representing only 50% of the population. In disease models where a decrease in activity is a measure of phenotype expression, such as Huntington's disease, it is important to measure activity consistently in an undisturbed home cage, and over more than one light/dark cycle, to ensure detection of subtle and progressive changes in phenotype (Figs 1, 2 and 3). For example, in disease models such as ALS and Huntington's disease, researchers may need to focus on particular times of increased baseline activity, such as the transition between the light and dark phases, to be able to detect biologically relevant parameters. This is particularly the case for males, which have a lower baseline activity level than females. Figure 3C illustrates this for data from the period of high voluntary activity during the last 30 min of darkness (active phase), where it is possible to detect decreased activity, measured as distance travelled or cage‐lid‐climbing activity, of HD mice as early as 8 weeks of age.

### (2) Exploration

Animals explore their environments to gather information (e.g. presence of predators or competitors) or look for resources (e.g. food or mates). Observing the exploratory behaviour of mice and rats can provide valuable insights into their cognitive processes, affective states and behaviour, all of which are important in research on neuroscience, medicine, and psychology (Novak *et al*., 2015). Like other animals and humans, laboratory rodents learn from previous experiences in similar situations to choose the best option when faced with a decision. In uncertain situations, this decision‐making process usually involves weighing two competing priorities: exploitation and exploration. Exploitation involves choices of options that are expected to be rewarding, and exploration involves the investigation of alternatives that might be more valuable. The expression of exploratory behaviour has been reported to differ between the sexes for many mental disorders (Chen *et al*., 2021). Chen *et al*. (2021) tested male and female mice for several days using a classical exploration/exploitation test: a restless unsteady two‐armed bandit task. Male mice made more exploratory decisions overall, while females explored less but learned faster during exploration, suggesting that sex has a stronger influence on decision‐making during the learning and exploration phases than during the stable, exploitative phases of decision‐making.

Social hierarchy status is an important component of the social behaviour of mice. Male and female social dominance hierarchies have a similar structure, but have different effects on overall behaviour. This can be studied using video‐based monitoring of home‐cage behaviour, a technique that eliminates artefacts created by a novel experimental environment, the presence of experimenters, or the presence of novel stimuli. Karamihalev *et al*. (2020) monitored the social dominance of males and females living in same‐sex groups in the enriched environment of the Social Box apparatus over a period of several days using the automated ANY‐maze video tracking system. Social dominance of males was found to be related to overall locomotion and entropy of exploration, suggesting territorial or patrolling behaviour. The study also suggested that social dominance influences the perception of and response to chronic stress differently in males and females (Karamihalev *et al*., 2020).

A Home‐Cage Rack and HCS high‐throughput real‐time option software were used by Burand Jr *et al*. (2023) to monitor a genetic rat model of Fabry disease for sex‐specific differences in exploratory behaviour associated with ongoing pain. Female rats showed significantly reduced home cage exploratory behaviour, moving and exploring less than male rats (Burand Jr *et al*., 2023).

### (3) Learning

As described in Section III.2, sex has a significant impact on decision‐making processes during the exploration and learning phases (Chen *et al*., 2021). In addition, research on gene activity in the brains of male and female mice revealed more than 1,000 differences in gene activation between the two sexes, which could influence sex‐typical behaviour and learning processes (Goldman, 2022).

**Fig. 4:**
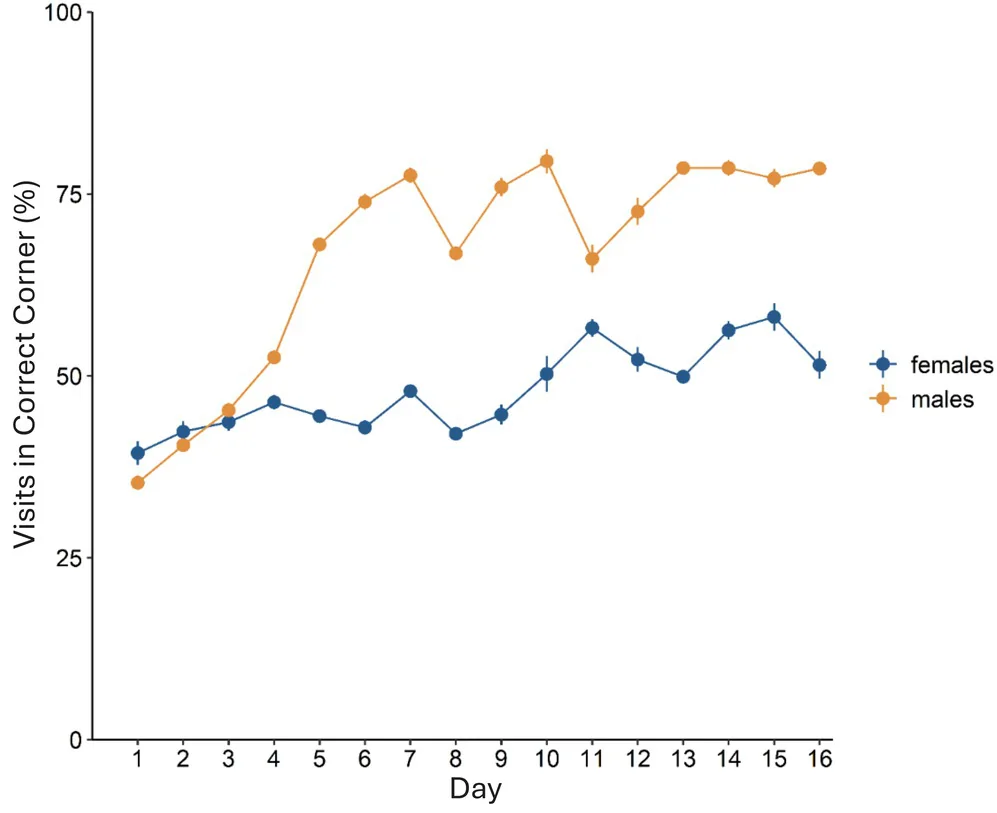
Complex spatial learning in the IntelliCage system. Male C57Bl/6 mice were more effective at a learning task than females, achieving over 50% correct responses on day 4 of learning and consistently exceeding 75% by day 13 (*P* < 0.001, two‐way ANOVA with repeated measures). Error bars are ±1SEM (*N* = 6 females throughout; for males, *N* = 7 to day 9, *N* = 6 for days 10–16; see Data S2). Percentage visits in correct corner were calculated as correct visits/all visits. Data are from unpublished data from Anna Kirk (available in Data S2).

In some tasks, female laboratory rodents have demonstrated better learning abilities than males. This was shown in the fear conditioning task in rats (Pupikina & Sitnikova, 2023), and in reward learning within a value‐based decision‐making task using a restless two‐armed bandit in mice (Chen *et al*., 2021). However, when examining other complex hippocampus‐dependent tasks in the social environment of the IntelliCage system, an opposite trend waas found: male mice showed better learning abilities than female mice (Fig. 4; see Data S2). Additionally, early‐life stress has been shown to affect the cognitive abilities of female mice disproportionately. Females subjected to early‐life stress showed impaired rule‐reversal learning, while stressed males did not (Goodwill *et al*., 2018). Further analysis of the brain revealed reduced expression and density of interneurons expressing parvalbumin in the orbitofrontal cortex of females, and the authors suggested that this caused the observed sex differences in cognitive impairment (Goodwill *et al*., 2018).

### (4) Emotions (aggression, depression, anxiety)

The emotional lives of men and women differ in several important ways. In general, women tend to experience prosocial emotions such as empathy, compassion, and parental love more strongly than men, who in turn tend to experience antisocial emotions such as anger, shame, and contempt more intensely than women (Schirmer, 2013). Aggression is one of the most important sex‐specific emotional social behaviours. In both sexes, aggression has evolved as an adaptive strategy to mediate competition for limited resources (e.g. nutrients, territory, and mating opportunities) that are essential for survival and reproductive success. In predominantly polygynous mammals, male aggression tends to be higher than female aggression, which may allow males to maximise their reproductive success (Gray, 1971; Oliveira & Bakker, 2022). Aggression in mammalian females often increases before or during lactation to protect their offspring and reduce the risk of infanticide (Duque‐Wilckens & Trainor, 2017). As a result, most neurobiological studies of aggressive behaviour have focused primarily on males or lactating females. However, recent reviews of research in humans (Denson *et al*., 2018) and rodents (Oliveira & Bakker, 2022) have shown that non‐lactating females can exhibit aggressive behaviour as frequently as males or lactating females. In humans, both sexes can display various forms of maladaptive aggression, such as conduct disorder, antisocial personality disorder, or intimate partner violence. Such persistent aggressive behaviour can cause serious psychological harm to those around them. For example, both men and women who have experienced intimate partner violence are at risk of developing post‐traumatic stress disorder, depression, anxiety, and suicidal thoughts (Ahmadabadi *et al*., 2020; Ludermir *et al*., 2008; Scott‐Storey *et al*., 2023). Aggressive behaviour can also emerge in individuals suffering from depression or social anxiety disorder (Zhang *et al*., 2023).

A variety of rodent models have been used to investigate the mechanisms underlying natural and pathological human aggression. Based on the assumption that males are more aggressive than females, most conventional tests have historically been standardised for males. Aggressive behaviour can be categorised into two primary types: reactive and appetitive aggression. Reactive (defensive) aggression can be assessed using the resident–intruder test, in which an intruding male can interact freely with a resident male in the resident's home cage. Appetitive (offensive, rewarding) aggression, on the other hand, is assessed using the conditioned place preference (CPP) test, in which a male can exhibit context‐dependent aggression toward a naïve male. To compare reactive and appetitive aggression between the sexes, two research teams (Aubry *et al*., 2022; Börchers *et al*., 2023) recently employed automated HCM systems to demonstrate interspecific differences in rewarding aggression in outbred mouse (CFW) and rat (Sprague–Dawley) strains. Aubry *et al*. (2022) used a video‐recording system for the resident–intruder test and a CPP test device to assess reactive and appetitive aggression in CFW mice housed with opposite‐sex cage mates. While males were more likely to engage in fighting, explored less, and found aggression rewarding, females were more likely to bite and alternated between aggression and exploration without perceiving aggression as rewarding or reinforcing. Börchers *et al*. (2023), by contrast, recorded the movements and aggressive behaviour of same‐sex Sprague–Dawley rats in the CPP test using a camera mounted above the arena and tracked with EthoVision XT 13. They also measured mesolimbic dopamine turnover after resident rats won an aggressive encounter. Unlike the CFW mice, both male and female rats perceived winning aggressive encounters as rewarding and exhibited aggression‐seeking behaviour. Residents that won an encounter showed increased accumbal dopamine turnover, suggesting that dopamine actively mediates aggression reward in rats.

Emotional disturbances are characteristic of affective disorders such as depression and anxiety. It is well established that women are approximately twice as likely as men to suffer from these disorders (Altemus, 2006; Madigan *et al*., 2023; Liu *et al*., 2024). Although rodent models are routinely used in preclinical research on affective disorders, findings have been inconsistent: some studies reported greater depression‐like behaviours in males (Kokras & Dalla, 2014; de la Rosa *et al*., 2024), others in females (Tao *et al*., 2024; Goodwill *et al*., 2019), while still others observed no sex differences at all (Moore, Kennedy & Hassan, 2024). Moreover, sex‐dependent outcomes appear to vary with the type of stress model (Iqbal *et al*., 2020), as well as with endophenotype and strain (Pitzer, Kurpiers & Eltokhi, 2022). This variability in results is influenced by multiple factors, including strain, experimental methodology, and the age of the subjects. In contrast to humans, where depression is more prevalent in females, depression‐like behaviours are often observed in male rodents (Heinsbroek, Vanhaaren & Vandepoll, 1988; Pitzer, Kurpiers & Eltokhi, 2022). This may result from a strong male sampling bias and/or sex differences in baseline activity in laboratory rodents during behavioural tests for anxiety and depression. Using a combination of modern methodologies, including a new, stable model of depression induced by chronic, unpredictable, mild stress, the SuperMaze behaviour analysis system, and VisuTrack animal behaviour analysis software, Xia *et al*. (2023) recently demonstrated that female Sprague–Dawley rats can indeed exhibit more depression‐like behaviour than their male counterparts.

An individual's sex and social status are decisive factors in determining the risk of developing anxiety or depression under conditions of chronic stress. Karamihalev *et al*. (2020) used an automated high‐throughput behavioural monitoring system to investigate the effects of social environment on interindividual variability in behavioural responses to chronic stress in same‐sex, group‐housed adult ICR/CD‐1 mice. Using video cameras mounted above the social box and a custom‐built automated tracking system, they determined the location of individual mice over time. Agonistic interactions derived from the location data revealed that, unlike males, which established a stable social hierarchy, only the highest‐ranking females maintained social stability. Subordinate males exhibited more anxious behaviour in stressful situations, whereas dominant females were bolder and less anxious. It is important to emphasise that postnatal development of anxiety and depression can also be influenced by social and psychological stressors in early life. To assess the effects of early‐life stress on depression‐like phenotypes, Goodwill *et al*. (2019) exposed newborn C57BL/6N mouse pups to fragmented maternal care induced by restricted bedding during lactation and continuously video‐monitored their behaviours – including grooming, resting, and walking – in a novel home cage for 5–11 days during early adolescence and young adulthood. They found that early‐life stress induced an early‐onset, female‐specific depressive phenotype, in which the females groomed less, rested more, and walked less than females reared under control conditions.

### (5) Social interactions, vocalisation

Sociability (i.e. an individual's willingness and ability to engage in social interactions with others) is an evolutionarily conserved trait among mammals (Clutton‐Brock, 2021). However, its expression and underlying neural and motivational mechanisms have been independently shaped in humans (Adolphs, 2003) and social rodents (including mice and rats; Kondrakiewicz *et al*., 2019) under distinct selective pressures. Women are generally considered to be better at empathising and socialising than men (Proverbio, 2023). Sex differences in impaired social functioning contribute significantly to sexual dimorphism in the development of many mental disorders, such as ASD, personality disorders, schizophrenia, and social anxiety disorder. Conversely, sex differences in the prevalence of these disorders may partly reflect sexual dimorphism in social rewards (Granza *et al*., 2023). The most rewarding and sexually dimorphic social interactions in laboratory rodents are aggressive and reproductive behaviours, including mating and parental care (Zilkha *et al*., 2021). As shown in both rats (Bartal, Decety & Mason, 2011) and mice (Scheggia *et al*., 2022), males and females can also differ in terms of their altruistic, prosocial behaviour.

Paradoxically, however, sensitivity to social reward and the recognition of emotions in conspecifics have been studied mainly in male rodents. To address this knowledge gap, Misiolek *et al*. (2023) and Granza *et al*. (2023) recently investigated prosocial choices, sensitivity to social reward, and the ability to recognise the affective state of another individual in adult male and female C57BL/6 mice. Using a specially designed maze, Misiolek *et al*. (2023) showed that only female mice increased the frequency of prosocial choices towards a familiar sibling partner during the experiment. By contrast, using EthoVision XT 15 software to analyse video‐recorded behaviour automatically in the CPP and affective state recognition tests, they found no effect of the sex of the animal on its sensitivity to social reward or the affective state of a same‐sex conspecific. By contrast, Granza *et al*. (2023) used a video‐monitored CPP three‐chamber apparatus and ANY‐maze video tracking software to examine sex differences in social reward in the C57BL/6J mouse sub‐strain. Unlike Misiolek *et al*. (2023), they found same‐sex social interactions more rewarding for adult males than for females.

During social interactions, individual mice and rats primarily communicate with conspecifics by emitting ultrasonic vocalisations (USVs; fundamental frequency range ~20–95 kHz) (Venkatraman *et al*., 2024). These USVs are species‐, strain‐, age‐, sex‐, context‐, and affective state‐specific in terms of rate, duration, and spectral characteristics. USV categorisation is increasingly used as a biomarker for qualitative changes in rodent vocalisations in models of mental disorders, including ASD (Caruso, Ricceri & Scattoni, 2020). Researchers therefore employ a range of complementary automated ultrasound detection and analysis methods to understand the complexity of USVs across specific life stages and contexts.

Sex differences in USVs are detectable from early life. To assess bidirectional dyadic mother–pup communication during lactation, Winters *et al*. (2023) developed a new automated home‐cage behavioural method [the Bidirectional Automated Mother‐pup Behavioural Interaction test (BAMBI)] using an ultrasound microphone, Avisoft SASlab Lite software, a Foscam C2 IP camera, and frame‐precision Shotcut software. The authors observed that male C57BL/6J mouse pups emitted USVs of shorter duration than their female counterparts. However, pup sex did not affect maternal retrieval.

Two studies have investigated the influence of sex and genetic background on the adult vocal repertoire. Zala *et al*. (2017) used a condenser ultrasound microphone mounted inside a recording chamber to demonstrate that wild‐derived mice of both sexes vocalised more frequently and at a faster rate during opposite‐sex than same‐sex interactions. Caruso *et al*. (2022) recorded vocalisations from male–female and female–female pairs in three strains (outbred CD‐1/ICR and inbred C57BL/6 and FVB mice) using a standard ultrasonic microphone mounted above the cage floor. In interactions with sexually receptive females, FVB males vocalised more frequently than C57BL/6 and CD‐1/ICR males. Similarly, female FVB mice vocalised more frequently than female C57BL/6 and CD‐1/ICR mice during interactions with other females.

However, it is not possible to associate a vocalisation with an individual or to detect low‐frequency vocalisations (LFVs; 4–10 kHz) with a standard microphone placed in the arena. To address this limitation, John *et al*. (2023) proposed a novel method utilising a small ultrasound microphone (Avisoft) inserted into the subject's nasal cavity. Using this microphone, together with a Flea3 USB3 camera and automated software, they studied sex differences in individual USVs and sniffing patterns during free and restricted social interactions in adult Sprague–Dawley rats. During interactions with female rats, males emitted significantly more USVs than during interactions with male rats. Conversely, female rats emitted more USVs in the presence of another female than in the presence of a male. Furthermore, female rats produced more USVs in same‐sex interactions than male rats did. Females also consistently emitted more LFVs than males during interactions with male rats.

### (6) Maternal care

In altricial rodents such as mice and rats, the full expression of maternal behaviour is crucial for survival, successful development, and future reproductive prospects of the dependent offspring, as well as for the health of the mother (August *et al*., 2021; Orso *et al*., 2018; Weber & Olsson, 2008). The behaviour of lactating female mice and rats is influenced by a number of factors, including genetic variants (Champagne *et al*., 2007), environmental and social conditions (Orso *et al*., 2019), maternal behaviour (Champagne, 2008), and offspring sex (Beery & Francis, 2011).

Wild mice and rats exhibit a polygynous mating system in which maternal investment generally has a greater effect on male reproductive fitness than on female reproductive fitness (Trivers & Willard, 1973). It is assumed that under favourable conditions, females of such species invest more in sons than in daughters. This theoretical expectation has also been confirmed in laboratory rodents. For example, Moore & Morelli (1979) found that mothers of Long‐Evans rats lick the anogenital region of male pups more frequently than that of female pups. Hao *et al*. (2011) used a video‐recording system to show that mother Sprague–Dawley rats licked the anogenital region and other body areas of male pups more frequently than those of female pups. This sex‐biased maternal care is crucial for sexual differentiation of the brain and for the development of normal sexual behaviour in both sexes (Beery & Francis, 2011).

Rodent models have long been used in biomedical research to explain the effects of environmental factors on the developmental programming of offspring (Orso *et al*., 2019; Souza, Moura & Lisboa, 2022). Offspring development is known to depend on maternal care and environmental conditions during gestation and lactation. Knowledge of the mechanisms underlying such maternal effects is crucial for understanding the developmental origins of health and disease (DOHaD). To highlight the influence of prenatal and perinatal environmental conditions on the development of human disease in adulthood, Hanson & Gluckman (2014) proposed the DOHaD hypothesis, according to which early environmental exposure can have long‐term effects on an individual's physiology. Many of these effects are sex specific and are linked to a wide range of mental illnesses and disorders. While women and girls are reportedly more likely to develop eating disorders, panic attacks, post‐traumatic stress disorder, major depression, social and generalised anxiety, phobias, Alzheimer's disease, and dementia, men and boys are reportedly at higher risk of ASD, attention deficit hyperactivity disorder (ADHD), Tourette's syndrome, Parkinson's disease, antisocial personality disorder, and substance abuse (Kudielka & Kirschbaum, 2005; Palanza & Parmigiani, 2017).

Sex‐specific effects of maternal care on offspring behaviour and health have been studied using HCM and rodent models in three important protocols: (*i*) pup retrieval; (*ii*) maternal separation; and (*iii*) litter size reduction.

The *Pup Retrieval test* is the most widely used test to assess maternal care and offspring development in laboratory rodents. It is employed to evaluate the effects of pharmacological and environmental interventions on maternal care and serves as a model for studying various neuropsychiatric disorders (including ASD, ADHD, obsessive‐compulsive disorder, anxiety, postpartum depression, and schizophrenia), foetal alcohol syndrome, and chronic prenatal stress, all of which can affect mother–infant interactions (Carcea *et al*., 2021; Ponchio *et al*., 2015). Although this test is commonly used to assess maternal behaviour in laboratory rodents, current knowledge of the potential importance of pup sex on maternal retrieval behaviour is limited. To investigate the relationship between pup sex and maternal retrieval behaviour, Winters *et al*. (2023) examined the responses of C57BL/6J mouse mothers to USVs of their pups, using synchronised behavioural and audio data recordings with a Foscam C2 IP camera and an ultrasonic microphone in the home cage at five time points during lactation. They found that, although male pups emitted shorter USVs than female pups, maternal retrieval behaviour was not influenced by pup sex.

The *Maternal Separation model* is routinely used to induce a variety of health‐related problems, including a disrupted mother–infant relationship (Orso *et al*., 2018), reduced or increased maternal care (Aguggia, Suarez & Rivarola, 2013; Orso *et al*., 2019), maternal stress responses and memory impairments (Aguggia *et al*., 2013), chronic early‐life adversity (Baracz *et al*., 2020), adverse brain development and impaired stress resilience (Čater & Majdič, 2022; Nishi, Horii‐Hayashi & Sasagawa, 2014), anxiety and depression (Bolukbas, Mundorf & Freund, 2020), and altered gene expression as well as neurobiological, reproductive, and behavioural phenotypes (Nishi *et al*., 2014; Orso *et al*., 2018). White *et al*. (2020) used a Med Associates fear‐conditioning chamber and Video Freeze software to show that unpredictable postnatal stress involving maternal separation in BALB/cByj mice reduced contextual freezing behaviour in adult male, but not female, offspring. This sex‐specific difference was mediated by changes in fronto‐limbic connectivity between the amygdala and the ventral hippocampus. The work of Gapp *et al*. (2014), in which C57BL/6 mice were automatically tracked by Viewpoint and tested in IntelliCages, provides a clear example of maternal separation‐induced sex‐specific transgenerational effects. Exposure of newborn mice to unpredictable maternal separation and maternal stress decreased expression of the mineralocorticoid receptor gene in the hippocampus and enhanced goal‐directed behaviours and reversal learning in the adult male offspring, suggesting that adverse early‐life experiences can sometimes produce adaptive transgenerational effects.

The *Litter Size Reduction model* is a useful tool to study the role of maternal care and postnatal overfeeding (PNOF) in DOHaD (Souza *et al*., 2022). It mimics early overfeeding and effectively models the development of obesity and its associated metabolic disorders, including type‐2 diabetes, non‐alcoholic fatty liver disease, cardiovascular disease, chronic kidney disease, and cancer (see Yang, Liu & Zhang, 2022). In addition, PNOF in young mice can exert transgenerational effects across two subsequent generations, including glucose intolerance in adulthood (Pentinat *et al*., 2010). Beyond these effects, PNOF induces adaptive epigenetic changes that lead to increased hypothalamus–pituitary–adrenal (HPA) axis activity and enhanced glucocorticoid sensitivity in adulthood. These epigenetic mechanisms include increased expression of glucocorticoid receptor genes (Chourbaji *et al*., 2011), which may subsequently promote the development of neuropsychiatric disorders, including anxiety and depression. The effects of PNOF on offspring phenotype have been studied mainly in males. However, exposure to PNOF can induce obesity phenotypes in both sexes. To elucidate the mechanisms underlying the long‐term effects of PNOF, Li *et al*. (2013) used the Comprehensive Laboratory Animal Monitoring System (CLAMS) and calorimetry home cages in FVB/NJ mice reared in control or small litters. They observed sex‐specific developmental, behavioural, and metabolic effects, with PNOF masculinising central nervous system signalling pathways and reducing spontaneous activity and energy expenditure in adult female mice from small litters.

### (7) Motivation

Motivation can also be assessed in the context of monitoring certain behaviours in drug addiction that are known to be sex dependent (Carroll & Anker, 2010). In animal models, sucrose‐sweetened foods or beverages are often used as high‐level reinforcers (Avena, 2007). However, operant reinforcement models are used to investigate and understand reinforcer‐driven behaviour more precisely. Response rate to a sucrose lever using a progressive schedule of reinforcement can indicate motivation to acquire the reinforcer (Richardson & Roberts, 1996). Sex differences in reactivity to sucrose cues have been reported. Female rats developed more Pavlovian approach behaviour to a sucrose‐paired stimulus compared to male rats (Cox *et al*., 2013), with an overall stronger motivation of females to consume sucrose being demonstrated (Grimm *et al*., 2022).

Gapp *et al*. (2014) showed that bad experiences could influence motivational behaviour across generations, as postnatally stressed male mice produced offspring with altered goal‐directed behaviours and behavioural flexibility. They exposed newborn C57BL/6 mice (F1 generation) to unpredictable maternal separation and stress for 2 weeks. Adult stressed F1 males were then used to produce the next generation of offspring (F2 generation) with wild‐type females. Stressed mice of both sexes and both generations were found to have reduced aversion and avoidance in the Elevated Plus Maze. Females were then tested for response under non‐aversive conditions using a delay‐discounting test in IntelliCages. The animals chose between a non‐responsive but immediate reward (water) and a responsive but delayed reward (saccharin). In addition to choice strategy, this test also assessed impulsivity. Stressed F2 females showed better goal‐directed behaviour compared to control females. Saccharin consumption was the same in stressed and control females, so choice was not due to an increased preference for saccharin. Next, females were tested for behavioural flexibility using a behavioural sequencing task in IntelliCages. Stressed F2 females showed better behavioural flexibility and better adaptation to novel rules than the control females. This effect of stress on behavioural flexibility has also been confirmed in males using a differential reinforcement lower rate paradigm that does not require group housing. Stressed males of both generations showed better goal‐directed behaviours than control males. The researchers concluded that the improved behavioural flexibility in males and females was not due to altered learning, as they also tested the animals with an active avoidance test and found no difference between control and stressed mice. The stress‐induced behavioural changes, which apparently also appear to be transmitted to the offspring, led to epigenetic changes in the hippocampus.

### (8) Feeding and drinking

Feeding and drinking are multifactorial behaviours essential for growth, body mass regulation, and survival. Preclinical studies of these fundamental behaviours in rodent models are particularly important for understanding the aetiology of health disorders. Because female rodents were long thought to be strongly influenced by hormonal fluctuations during the oestrus cycle, food and water intake and their behavioural effects were traditionally studied only in males (Christians, Shergill & Albert, 2021). Although food and water availability can influence the phenotype and health of both sexes in similar ways (e.g. Souza *et al*., 2022), certain outcomes nevertheless show consistent sex differences (Fukushima *et al*., 2015; Mort *et al*., 2023; Santollo & Edwards, 2021).

The HPA axis plays an important role in the development of eating disorders in humans (Lo Sauro *et al*., 2008). Chronic exposure to glucocorticoids such as cortisol and corticosterone can contribute to the development of behavioural, metabolic, nutritional, and neuropsychiatric disorders (Sheng *et al*., 2021). Moreover, stress‐related negative emotional states can trigger emotional eating and the consumption of palatable foods, which may in turn promote obesity (Dakanalis *et al*., 2023). In mammals, females generally show a stronger HPA response than males (Larkin *et al*., 2010). Consequently, females are at higher risk of developing stress‐related disorders such as major depression or generalised anxiety disorder (Hodes, 2013). These sex differences are further reflected in women's greater susceptibility to eating disorders (Altemus, Sarvaiya & Epperson, 2014). In addition, women with obesity are more likely than men to develop depression at a younger age (Maeng & Milad, 2015). Since E2 and T reduce adiposity, whereas high progesterone levels promote it, sex‐specific effects of diet on emotional behaviours may be mediated by gonadal hormones (Clark *et al*., 2022).

Sex‐specific differences in eating behaviour are well illustrated by three recent rodent studies showing sexual dimorphism in the development of obesity (Maric *et al*., 2022) and addictive behaviour (Grimm *et al*., 2022; Wei *et al*., 2021). Maric *et al*. (2022) investigated sex differences in diet‐induced obesity evaluated in the home cage, in laboratory mice and rats in their home‐cages and found that, in both species, diet‐induced hyperphagia was greater in males, whereas daily consumption of a high‐fat diet (HFD) was greater in females. However, the effects of sex on HFD‐induced changes in body mass gain and metabolism differed between species. Female Sprague–Dawley rats fed an HFD showed delayed diet‐induced body mass gain and fewer metabolic complications than males, whereas C57BL/6 mice of both sexes fed an HFD developed an obese phenotype and impaired glucose tolerance. By contrast, to explore sex differences in sucrose‐based addictive behaviour, Wei *et al*. (2021) and Grimm *et al*. (2022) developed preclinical models of sucrose consumption in C57BL/6N mice and Long‐Evans rats, respectively. Wei *et al*. (2021) used the Drinkometer system placed in the home cage together with operant chambers, which allowed precise quantification of sugar solution intake. Female C57BL/6N mice were more prone than males to excessive sugar consumption, craving, and relapse (Wei *et al*., 2021). Similarly, using operant conditioning chambers, Grimm *et al*. (2022) found that female Long‐Evans rats were more motivated to consume sucrose, ingested larger amounts, and responded more quickly for sucrose rewards than males. No significant sex difference was observed in saccharin preference in this strain (Grimm *et al*., 2022).

The drinking patterns of male and female rodents have been described mainly in relation to alcohol, sucrose, and other liquids rather than water. Sex‐dependent differences have been observed in both preferences and the extent of alcohol consumption in the home cage. When alcohol was available, both male and female mice preferred it to water. However, females consistently consumed more alcohol per unit body mass than males (Rivera‐Irizarry *et al*., 2023). Importantly, this increased alcohol intake in females was not due to a general rise in fluid intake but was specific to alcohol. This suggests that female rodents prefer alcohol to water when given a choice, highlighting a sex‐dependent pattern of drinking behaviour in both mice (Rivera‐Irizarry *et al*., 2023) and rats (Foo *et al*., 2023). The higher alcohol consumption in females has been linked to hormonal fluctuations, body composition, and neural mechanisms such as reward network wiring. Serotonin 5HT2c receptor‐expressing neurons and oestrogen signalling in the dorsal raphe of the brain may play a role in regulating alcohol consumption in females (Flanigan *et al*., 2023; Torres Irizarry *et al*., 2024). Both male and female mice showed a strong preference for sucrose solution over water (Wulff *et al*., 2023), supporting the tendency towards sugar addiction.

Water consumption tends to decrease with age. In humans, older adults drink less water, primarily due to a decrease in thirst, which may be influenced by the increased prevalence of chronic diseases and the use of multiple medications (Pence *et al*., 2025). However, in rodents this phenomenon has been examined mainly under thirst challenges, such as water deprivation or osmotic load, and age‐related changes in spontaneous water intake under standard conditions remain poorly studied.

## IV. TRANSLATIONAL PRECAUTIONS

The transfer of findings on sex‐specific behaviours and activities from rodent models to humans poses several challenges. First, the biological and environmental differences between rodents and humans are likely to lead to discrepancies (Premachandran, Zhao & Arruda‐Carvalho, 2020). Second, rodent behaviour is influenced by their immediate survival needs, which can differ significantly from human behaviour. Third, the interpretation of rodent behaviour is often subjective if an automated monitoring system is not used and can vary depending on the observer's perspective (Colom‐Lapetina *et al*., 2019). Finally, human sociocultural influences add a layer of complexity that is not present in rodent models (McGregor *et al*., 2016). Therefore, while rodent models provide valuable insights, careful interpretation, and validation of results in human populations is essential.

## V. ETHICAL AND THREE RS IMPLICATIONS OF SINGLE‐SEX STUDIES

The ethical and Three Rs (the principles of Replacement, Reduction and Refinement of animal use) implications of single‐sex studies are seldom explored. While some researchers (Neville, Lecorps & Mendl, 2022) have wrongly stated that the use of both sexes would be against the Three Rs principle of Reduction, this typically arises from several misconceptions. The first misconception regards the meaning of Reduction, which in its original formulation was deemed as ‘reduction in the numbers of animals used to obtain information of a given amount and precision’ (Russell & Burch, 1959, p. 64), i.e. by not only obtaining equivalent information with fewer animals but also by getting *more* information using the same number of animals. Single‐sex studies achieve neither. This leads to the second misconception, which is that, in order to use both sexes, twice as many animals are necessary. However, a simple statistical power calculation can easily show how factorial designs allow using the same overall number of animals with negligible power loss (Franco & Fry, 2023). Moreover, an experimental design and analysis approach allows detection of sex × treatment interactions, which would not be possible when studying only one sex and, when no interactions exist, factorial designs have higher test sensitivity and statistical power. Furthermore, in the specific case of HCM, repeated measurements within the same animal increase the precision of estimates and can enhance statistical power. This design means that, when analysed with appropriate repeated‐measures models, it is often possible to detect effects with fewer animals than would be required in single‐measure designs (Voikar & Gaburro, 2020). The third misconception is the incorrect belief that using female animals would lead to higher variability, and hence to less‐reliable results and possibly the use of more animals to counter the increased variability. However, results from HCM studies show that this is not the case regarding behaviour. Shansky (2019) showed that preference for males on the basis of higher hormonal variability of females is unfounded, and warned against an approach of using males first, and then testing on females if results were interesting. This perception of males as the ‘default sex’ in experiments has clinical, social, and ethical implications (Zhao *et al*., 2023). Furthermore, not only does this approach have inherent bias by making testing on females contingent on results in males, but also there is no way of ruling out an influence of variation in experimental conditions (litter, environment, husbandry, etc.) between two studies conducted at different times. There are examples where males have been excluded as experimental subjects (Franco *et al*., 2012), as is often the case in long‐term studies, likely to avoid the higher intra‐group aggression observed in males. However, such behaviour can be mitigated through good husbandry (Weber *et al*., 2022), and in any case should not preclude the use of animals of both sexes.

Testing one sex at a time also leads to using twice as many animals as necessary, and possibly to the killing of up to four times as many since, for each single‐sex study, animals of the non‐used sex may be needlessly culled. Another ethical implication of wasteful research is its impact on the animal care staff, as the wasteful killing of healthy animals has been identified as a cause of compassion fatigue, and can impact staff empathy and their care for the animals (Thurston *et al*., 2021). There has been a recent heated debate in Germany on whether such waste amounts to a crime (Feldwisch‐Drentrup, 2022). Whether or not this is true, wasteful single‐sex studies are undesirable from both an economical and ethical viewpoint (Franco, 2016; Meijboom & Stassen, 2016; Franco & Olsson, 2016). Factorial designs using both sexes prevent such waste, hence meeting ethical requirements, legal demands, and public expectations better (Olsson & Franco, 2015), while fostering a Culture of Care.

## VI. CONCLUSIONS

(1) Historical and ongoing biases towards single‐sex studies in neuroscience and biomedical research are scientifically ungrounded, skew research findings, and have significant social and ethical implications.
(2) Assumptions that behavioural variability in female rodents is higher than in males conflict with the available evidence, raising the issue of why most preclinical studies focus on animals of one sex, with a clear male bias.
(3) Automated Home‐Cage Monitoring systems allow for comprehensive naturalistic observations of rodent behaviour, revealing sex‐specific differences that may not be detected in conventional short‐term or out‐of‐cage tests.
(4) Factorial study designs that include both sexes are recommended, as they allow detection of both sex differences and sex × treatment interactions, without requiring an increase in the number of animals used.
(5) Addressing sex bias in animal studies can enhance the translational value of preclinical research by providing better information on the efficacy and safety of therapeutic interventions for the whole population.

## AUTHOR CONTRIBUTIONS

M. Č. conceptualised the manuscript, assembled the team, managed meetings and correspondence, and integrated all textual and visual contributions; M. Č., A. D., A. B., S. M., E. G., M. Rig., R. S. B., M. Riv., M. L., A. K., L. L. and N. H. F. wrote the text, and provided references and/or figures; L. L. provided updated bibliometric data on sex bias in HCM studies for 2022 and 2023; R. S. B., S. M., M. Riv., M. L., E. G., and A. K. provided data. H. F. reviewed the manuscript; N. H. F. performed the final critical review and editing, and coordinated its revision after peer‐review. All authors discussed the manuscript from the planning stage to its final form and contributed meaningfully to its content. All authors read and approved the final manuscript.

## Supporting information


**Data S1.** Data retrieved from Bains *et al*. (2023), Stolting *et al*. (2023), Rodriguez de los Santos *et al*. (2021) and the ABPF (unpublished data) showing distance travelled and time spent climbing per day.

**Data S2.** Raw behavioural data used in Fig. 4. The data include measurements of spatial preference performance expressed as the percentage of visits to the correct corner during a place‐learning task. Data are reported for laboratory mice, separated by sex and recorded across consecutive experimental days.

## Data Availability

Data shown in Fig. 1A,B top section are from Bains *et al*. (2023), available in Data S1 and at https://doi.org/10.3389/fnbeh.2023.1148172; data in Fig. 1A,B bottom level are from Stölting *et al*. 2023 (https://10.1172/jci.insight.162468), Rodriguez de Los Santos *et al*. (2021) (https://doi.org/10.1073/pnas.2014481118), and from unpublished data of control animals collected at the Animal Behavioural Phenotyping Facility, Charité Berlin, all made available as supplementary material (Data S1). Data shown in Figs 2A and 3B are from Golini *et al*. (2023), available at https://doi.org/10.3389/fnbeh.2023.1130055. Data shown in Figs 3A and 2B are from Golini *et al*. (2020), available at https://doi.org/10.3389/fnins.2020.00896. Data in Fig. 3B are from Golini *et al*. (2023). Data in Figs 2C and 3C are from Bains *et al*. (2023), available at https://doi.org/10.3389/fnbeh.2023.1148172. Data shown in Fig. 4 are from Anna Kiryk, and available as supplementary material (Data S2).
