# Supplementary material for: On the importance of including both sexes in animal studies – insights from home‐cage monitoring
Source: Biol Rev Camb Philos Soc. 2026 May 13;101(5):2408–26. doi: 10.1002/brv.70184 (PMC13542363; doi:10.1002/brv.70184)
Supplement: Supplementary file 1 [file BRV-101-2408-s001.docx]

Data S2. Raw behavioural data used in Fig. 4. The data include measurements of spatial preference performance expressed as the percentage of visits to the correct corner during a place-learning task. Data are reported for laboratory mice, separated by sex and recorded across consecutive experimental days.

| **Descriptors** | **Description** |
| --- | --- |
| Methodology | Data were collected during a behavioural experiment assessing visits to a predefined correct corner. For each experimental group and day, the mean percentage of visits to the correct corner was calculated based on the number of animals in the group. Variability is expressed as the standard error of the mean (SEM). |
| Variables | Group – experimental group designation  Day – experimental day number  Sex – sex of the animals (female or male)  Number of mice in group – sample size per group  Visits in correct corner (%) – mean percentage of correct corner visits  calculated as ratio of visits in correct corner to all visits  (correct+incorrect)*100%  SEM – standard error of the mean |
| Experimental subjects | C57Bl/6 mice, age 3–4 months, grouped according to sex. |
| Temporal coverage | Consecutive experimental days as indicated in the data set. |
| Author email | Contact of the main researcher: a.kiryk@nencki.edu.pl |

| **Group** | **Day** | **Sex** | **Number of mice in group** | **Visits in correct corner (%)** | **SEM** |
| --- | --- | --- | --- | --- | --- |
| control females | 1 | Female | 6 | 39,38% | 1,63% |
| control females | 2 | Female | 6 | 42,33% | 1,45% |
| control females | 3 | Female | 6 | 43,64% | 1,22% |
| control females | 4 | Female | 6 | 46,37% | 0,98% |
| control females | 5 | Female | 6 | 44,48% | 0,88% |
| control females | 6 | Female | 6 | 42,91% | 1,03% |
| control females | 7 | Female | 6 | 47,90% | 0,60% |
| control females | 8 | Female | 6 | 42,05% | 0,56% |
| control females | 9 | Female | 6 | 44,66% | 1,38% |
| control females | 10 | Female | 6 | 50,26% | 2,47% |
| control females | 11 | Female | 6 | 56,59% | 1,23% |
| control females | 12 | Female | 6 | 52,23% | 1,71% |
| control females | 13 | Female | 6 | 49,87% | 0,95% |
| control females | 14 | Female | 6 | 56,24% | 1,26% |
| control females | 15 | Female | 6 | 58,09% | 1,90% |
| control females | 16 | Female | 6 | 51,49% | 1,93% |
| control males | 1 | Male | 7 | 35,30% | 0,98% |
| control males | 2 | Male | 7 | 40,47% | 0,43% |
| control males | 3 | Male | 7 | 45,27% | 1,01% |
| control males | 4 | Male | 7 | 52,54% | 0,97% |
| control males | 5 | Male | 7 | 68,05% | 0,73% |
| control males | 6 | Male | 7 | 73,90% | 1,08% |
| control males | 7 | Male | 7 | 77,56% | 1,04% |
| control males | 8 | Male | 7 | 66,83% | 0,94% |
| control males | 9 | Male | 7 | 75,93% | 1,27% |
| control males | 10 | Male | 6 | 79,51% | 1,66% |
| control males | 11 | Male | 6 | 66,09% | 1,89% |
| control males | 12 | Male | 6 | 72,57% | 1,87% |
| control males | 13 | Male | 6 | 78,56% | 0,73% |
| control males | 14 | Male | 6 | 78,57% | 1,07% |
| control males | 15 | Male | 6 | 77,12% | 1,27% |
| control males | 16 | Male | 6 | 78,51% | 0,73% |
